# Navigating the Complexity of High-Risk Pulmonary Embolism—Is Mechanical Thrombectomy the Answer We Need?

**DOI:** 10.1016/j.jscai.2023.101178

**Published:** 2023-10-31

**Authors:** Hamid R. Mojibian, Khanjan Shah, Akhil Khosla

**Affiliations:** a Yale School of Medicine, New Haven, Connecticut; b University of Florida College of Medicine, Gainesville, Florida

**Keywords:** high-risk pulmonary embolism, massive pulmonary embolism, mechanical thrombectomy, pulmonary embolism

The treatment of high-risk or “massive” pulmonary embolism (PE) presents clinicians with a formidable challenge. Characterized by hemodynamic instability, rapid deterioration, and high mortality, high-risk PE is a medical emergency that mandates swift decision-making and effective therapeutic interventions. The conventional treatment paradigm of systemic thrombolysis and anticoagulation is currently the standard of care for first-line reperfusion therapy but is associated with high mortality and complications, namely bleeding. Moreover, there are numerous absolute and relative contraindications to systemic thrombolysis that nuances clinical decision-making.^1^

Recently, catheter-directed therapies, surgical thrombectomy, and mechanical circulatory support have all challenged the current paradigm of high-risk PE treatment. Catheter-based therapies offer the promise of localized clot disruption with minimized systemic bleeding risk.^2^ Rapid advances in catheter technology have propelled this field into an era of immense innovation and have potentially made mechanical thrombectomy (MT) an effective therapy with virtually no risk of systemic bleeding; however, one must remember that there is currently a lack of robust evidence, particularly randomized controlled trials, that leaves clinicians in somewhat uncharted waters. While observational studies and small trials suggest the efficacy and safety of catheter-directed therapies for intermediate-risk PE,^3^^,^^4^ we must exercise caution in extrapolating these results to a broader, sicker patient population.

In the current issue of *JSCAI*, Horowitz et al^5^ demonstrate that in a prospective cohort of 63 patients with high-risk PE enrolled in the FlowTriever All-Comer Registry for Patient Safety and Hemodynamics (FLASH) registry, MT with the FlowTriever System (Inari Medical) resulted in no major adverse events (MAE) following the procedure and 0% 30-day mortality.

Although promising, the critical questions we face today are not whether MT works but for whom it works best, under what circumstances, and how it compares with existing standards of care. As clinicians committed to advancing patient care through scientific rigor and innovation, we must dissect, scrutinize, and build upon such research to generate more comprehensive evidence. This call to action is not just an academic obligation but a clinical imperative that directly impacts patient outcomes.

## The patient population: Truly high-risk?

One of the focal points of the FLASH registry is its categorization of high-risk PE patients based on the European Society of Cardiology (ESC) guidelines.^1^ Patients who meet the criteria for high-risk PE using the ESC guidelines have considerable heterogeneity in severity of illness and acuity. For example, all the following patients are considered to be high-risk based on the current ESC guidelines: patients who require no to minimal vasopressors, patients who require multiple escalating doses of vasopressors or mechanical circulatory support, and those who have or are currently suffering a cardiac arrest. Given the significant variation in this population, multiple publications have raised awareness that additional risk stratification and treatment are needed for high-risk PE patients such as subclassifying high-risk PE as stable, unstable, and catastrophic.^6^

Despite meeting the ESC criteria for high-risk PE, less than half of the patients in the study by Horowitz et al^5^ ever required vasopressors either at presentation or at the time of MT; only 10% of patients required mechanical ventilation; and 6% suffered a cardiac arrest. Based on the information available, the majority of patients were relatively stable and may not represent the full spectrum of high-risk PE patients. While the cohort meets the criteria for high-risk PE and warranted reperfusion therapy as highlighted by the fact that 42.9% of patients had a depressed cardiac index <2 L/min/m^2^, an indicator of possible cardiogenic shock, and ∼13% of patients failed a prior treatment strategy, it may not represent the most unstable patients in clinical practice.

## Primary and secondary outcomes: What do they mean for the future?

The FLASH registry’s primary end point focused on MAE within 48 hours after procedure. Impressively, no patients met this composite primary end point, suggesting that the FlowTriever System is safe for high-risk patients. The safety profile should be emphasized given that systemic thrombolysis can be associated with major bleeding and sometimes fatal complications, which could be avoided with the use of MT if used as the primary reperfusion therapy. Secondary safety and effectiveness end points, including hemodynamic improvements and right ventricle/left ventricle ratio changes, were also promising. The study observed significant reductions in mean pulmonary artery pressure and systolic pulmonary artery pressure after procedure. It is still unclear if these surrogate end points translate into hard clinical end points including long-term mortality and the development of post-PE impairment. Importantly, a considerable number of patients did not require intensive care unit admission, which is generally required with other reperfusion therapies, suggesting that there may be an economic advantage.

The FLASH registry represents a seminal effort in capturing real-world data on the treatment of high-risk PE patients with MT methods like the FlowTriever System and sheds light on a potential alternative first-line therapy. This study focuses on MT as the primary reperfusion strategy, acknowledging that not all hospitals have access to this therapy and not all patients are stable for transfer to a procedure suite for such therapy. Given the impressive outcomes and safety results with MT and the fact that in this study it was used as both a primary treatment option and for those who had failed previous treatments, the question that arises is should all high-risk patients be treated with MT, and if not available, should patients be transferred to a center that can offer MT and other advanced therapies?

Future studies in the high-risk PE patient population will need to include all severities of illness for high-risk PE, assess time to reperfusion therapy, compare reperfusion therapies including combined approaches, and control for postreperfusion care. Perhaps most importantly, randomized controlled trials of MT compared with systemic thrombolysis are needed to answer the most basic question of do these devices make a difference clinically in patients with high-risk PE.

The uptake of MT for treatment of intermediate and high-risk PE has outpaced high-quality evidence, which opens the doors for physician bias and limits our ability to generate evidence due to lack of perceived equipoise. The high-risk PE patients enrolled in the FLASH registry demonstrate that we can successfully use MT as a first-line therapy with no MAE and a significant improvement in hemodynamic metrics. Given the persistent high mortality and complexity of high-risk PE, MT may be the answer we need to improve outcomes and move this field forward.
